# Characterization of Thermoresponsive Poly-N-Vinylcaprolactam Polymers for Biological Applications

**DOI:** 10.3390/polym13162639

**Published:** 2021-08-08

**Authors:** Lorenzo Marsili, Michele Dal Bo, Giorgio Eisele, Ivan Donati, Federico Berti, Giuseppe Toffoli

**Affiliations:** 1 Experimental and Clinical Pharmacology Unit, CRO National Cancer Institute, IRCCS, Via Franco Gallini 2, 33081 Aviano, Italy; mdalbo@cro.it (M.D.B.); gtoffoli@cro.it (G.T.); 2 Department of Chemical and Pharmaceutical Sciences, University of Trieste, Via Licio Giorgieri 1, 34127 Trieste, Italy; fberti@units.it; 3 Centro Alta Tecnologia “Istituto di Ricerche Chimiche e Biochimiche G. Ronzoni” Srl, via G. Colombo 81, 20133 Milan, Italy; eisele@cat-ronzoni.it; 4 Department of Life Sciences, University of Trieste, Via Licio Giorgieri 5, 34127 Trieste, Italy; idonati@units.it

**Keywords:** poly-N-vinylcaprolactam, thermoresponsive polymers, LCST

## Abstract

Poly-N-Vinylcaprolactam (PNVCL) is a thermoresponsive polymer that exhibits lower critical solution temperature (LCST) between 25 and 50 °C. Due to its alleged biocompatibility, this polymer is becoming popular for biomedical and environmental applications. PNVCL with carboxyl terminations has been widely used for the preparation of thermoresponsive copolymers, micro- and nanogels for drug delivery and oncological therapies. However, the fabrication of such specific targeting devices needs standardized and reproducible preparation methods. This requires a deep understanding of how the miscibility behavior of the polymer is affected by its structural properties and the solution environment. In this work, PNVCL-COOH polymers were prepared via free radical polymerization (FRP) in order to exhibit LCST between 33 and 42 °C. The structural properties were investigated with NMR, FT-IR and conductimetric titration and the LCST was calculated via UV-VIS and DLS. The LCST is influenced by the molecular mass, as shown by both DLS and viscosimetric values. Finally, the behavior of the polymer was described as function of its concentration and in presence of different biologically relevant environments, such as aqueous buffers, NaCl solutions and human plasma.

## 1. Introduction

Thermoresponsive polymers are characterized by a drastic and discontinuous change of their physical properties with temperature. Given a solvent/polymer binary mixture, the phase diagram usually exhibits a binodal curve that divides a polymer-rich zone from a zone in which the polymer and the solvent are miscible in every proportion. Accordingly, the critical solution temperature can correspond to the minimum or the maximum of the binodal curve. If the critical point corresponds to the minimum of the curve, the corresponding temperature is called lower critical solution temperature (LCST). On the contrary, the temperature corresponding to the maximum of the curve is called upper solution temperature (UCST) [1].

The ability to respond to a change in temperature makes thermoresponsive polymers a “smart” class of materials that can be applied in a broad range of applications [2]. To date, there are hundreds of thermoresponsive polymers developed for various applications in the biological field, which include tissue engineering, bioseparation, drug and gene delivery [2,3,4]. As a general rule, LCST-type polymers are easily solvated in water through hydrogen bonding and polar interactions [5]. Accordingly, their biological interest relies on the presence of a desirable sharp transition in aqueous systems [2]. Their utilization in the biological field requires that the LCST in aqueous solution is close to the normal human physiological temperature, which is usually comprehended between 36.5 and 37.5 °C, but can oscillate in a wider range between 33.2 and 38.2 °C [6].

PNVCL is a thermoresponsive polymer which exhibits LCST that has been applied in biomedical and environmental applications, in cosmetics and as an anticlogging agent in pipelines [3,4]. PNVCL is usually described as a biocompatible alternative to poly-N-isopropylamide (PNIPAM) [3,4,7,8,9,10,11,12,13,14,15] as both polymers can exhibit LCST behavior close to the physiological temperature. According to this misconception, PNVCL has been often described as a polymer with a well-defined LCST at 32 °C [7,8,9,16,17,18,19,20,21], even though the LCST has been observed in broad range of temperature between 25 and 50 °C [3,4]. As a matter of fact, PNVCL and PNIPAM exhibit a complete opposite miscibility behavior [3,22]. PNIPAM exhibits a LCST at about 32 °C, which is almost independent from the molecular weight (Type II) [3,4]. On the contrary, PNVCL miscibility can be described by the Flory–Huggins theory and depends on molecular weight [3,23], polymer, salt and protein concentration (Type I). Due to its molecular weight-dependent LCST, the control of both molecular weight and polydispersity in PNVCL synthesis is of crucial importance [3,4,22,23,24,25,26,27] for any application. Similarly, the determination of molecular mass must rely on appropriate methodologies that allow to correlate the synthetic procedures with the final properties of the polymer.

Although the thermoresponsivity of PNVCL was first observed in 1968 [23], the polymer is still introduced as a novelty in biological and nanoscientific publications [3,4,7,8,9,16,17,18,19,20,21]. The first “in vitro” evaluation of PNVCL cytotoxicity on intestinal Caco-2 and pulmonary Calu-3 cell lines was published in 2005. The test demonstrated great cell tolerance although PNVCL exhibited toxic effects above its LCST [28]. To date, the biocompatibility of PNVCL has been established in several different cell lines, including different types of human carcinomas [4,29,30,31]. PNVCL was also investigated as a suitable environment for cell proliferation and manipulation [32] and it is commercialized as a hair setting product under the name of Luviscol^®^ Plus [33]. The utilization of PNVCL with carboxyl end groups has been reported for the preparation of PNVCL-based delivery systems in conjunction with biocompatible polysaccharides, such as alginates [34], chitosan [7,9,16,17,18,19,20,21,35], or dextran [3,36,37]. A common approach is to use PNVCL-COOH polymers with a 32 °C LCST as starting materials for the preparation of biocompatible copolymers and thermoresponsive particles [7,8,9,19,35,38]. The LCST is raised according to the degree of substitution and the molecular mass of the polymer conjugated with PNVCL. However, to ensure reproducibility, PNVCL synthesis must be optimized in order to ensure control over chemical structure, molecular mass, polydispersity and morphology [3,4]. Moreover, it is still fundamental to assess the polymer thermoresponsive behavior in biologically relevant fluids such as human plasma.

The aim of this work is to rationalize this concept, as we considered the polymerization of different PNVCL polymers with carboxyl terminations (PNVCL-COOH) that exhibit different LCSTs according to their different molecular mass, their concentration and the solution environment. Furthermore, this work provides insight on different approaches for the determination of the molecular mass and the LCST of PNVCL. The polymers were prepared by using a modified approach of the protocol reported by Prabaharan [35] and reported by many different authors [7,8,9,16,17,18,19]. Accordingly, we reported the preparation of PNVCL-COOH polymers exhibiting LCST between 33 and 42 °C via FRP. The hypothesis of mass-dependent LCST was first supported by the comparison of ^13^C NMR spectra of different PNVCL-COOH, by assessing the variation of the signals related to the terminations as a function of the NVCL/AIBN ratio that were used for the synthesis. Finally, the determination of the molecular mass via viscosimetry and DLS demonstrated that the LCST of PNVCL-COOH is inversely proportional to the molecular mass. LCSTs were determined using UV-VIS and DLS at a concentration of 0.5 wt.%. The variation of LCST of PNVCL-COOH polymers was also assessed in relation to polymer concentration, the presence of NaCl and three different buffers: citrate (pH 3), acetate (pH 5) and phosphate (pH 7). Finally, we report a method for the evaluation of the polymer in human plasma. In particular, it was observed that it is possible to increase LCST by decreasing PNVCL-COOH concentration, while the presence of salts, particularly phosphates, results in a significant lowering of the LCST. The measurements performed in human plasma showed a relevant lowering of the LCST of PNVCL-COOH polymers by about 10 °C. These observations are particularly useful for physiological applications, as the polymers undergoing LCST may result in possible cytotoxic effects.

## 2. Materials and Methods

### 2.1. Materials

N-Vinyl Caprolactam (NVCL), 2,2′-Azobis(2-methylpropionitrile) (AIBN), 3-mercaptopropionic acid (MPA), were purchased from Sigma-Aldrich (St. Louis, MO, USA). N-hydroxy succinimide (NHS), Deuterium oxide (D_2_O), N,N′-Dimethylformamide anhydrous (DMF), Dimethyl sulfoxide-d_6_, Deuterium Chloride 37% (DCl/D_2_O) were purchased from Sigma-Aldrich (St. Louis, MO, USA). Spectra/Por^TM^ Cellulose membrane tubing (1–2 kDa, MWCO) was purchased from Thermo Fisher Scientific (Waltham, MA, USA).

### 2.2. Synthesis of PNVCL-COOH Polymers

**Figure 1:**
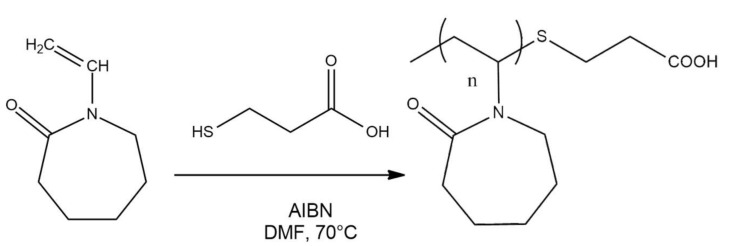
Synthesis of PNVCL-COOH polymers.

PNVCL-COOH was synthesized by free radical polymerization by using a modified version of the protocol reported by Prabaharan [35]. Prior to their utilization, NVCL was recrystallized in hexane and AIBN was recrystallized in ethanol, while MPA was transferred in a sealed vial that was deoxygenated with Ar. For all preparations, 5 mg of AIBN (0.304 mmol) and 28 µL of MPA (3.278 mmol) were used. Different molar ratios between NVCL and AIBN were used in order to obtain polymer with different molecular mass and LCST. The NVCL/AIBN molar ratio were, respectively, 122, 244, 305, 610, 1220 and 1690. DMF was deoxygenized with Ar for 15 min prior to the addition of the reagents (Figure 1). After the dissolution of the reagents, the reaction was carried out in sealed vials at 70 °C for 8 h. All sealed vials were dried overnight at 80 °C before the reaction. After the reaction, the solution was dialyzed in a cellulose membrane tubing (MWCO of 1–2 kDa) against distilled water for at least 2 days to remove impurities and unreacted materials. Finally, the frozen product was freeze-dried at −50 °C and 0.05 mbar and stored at 4 °C.

### 2.3. Structural Characterization of PNVCL-COOH Polymers

NMR spectra were acquired using a High-resolution 500 MHz Bruker NEOn500 Quadruple resonance (H/C/N/2H) equipped with a high-sensitivity TCI 5 mm CryoProbe (Bruker, Billerica, MA, USA) at 25 °C in D_2_O and d_6_-DMSO.

FT-IR spectra were recorded with a double-beam Perkin Elmer System 2000 Ft-IR Spectrometer (Perkin Elmer, Waltham, MA, USA) in the range of 4500–370 cm^−1^ using KBr pellets.

The number of terminal carboxyl groups was determined via conductimetric titration. The polymers were dissolved using diluted HCl and the solutions were titrated using a standardized solution of NaOH 0.1 M. The deprotonation of the -COOH end groups resulted in the formation of a small plateau in the conductimetric titration curve. The number of carboxyl groups was determined by the volume difference of added NaOH solution between the initial and the final point of the plateau (Figure S10).

### 2.4. UV-VIS Determination of LCST

Phase transition and absorbance measurements were carried out in a Shimazdu UV-visible spectrophotometer model UV-2450 equipped with a TCC-240A Thermoelectrically Temperature Controlled Cell Holder (Shimazdu, Kyoto, JP). Transmission data were used for the realization of the miscibility curves. The miscibility curves were fitted with a sigmoidal model in order to calculate the LCST as the inflection point by using the following equation:

(1) $$y=Tr_{max}+\frac{\left(Tr_{max}-Tr_{min}\right)}{1+e^{\frac{\left(T-LCST\right)}{dT}}}$$

where *Tr* is the calculated transmittance at a specific wavelength at a fixed temperature.

### 2.5. DLS Determination of LCST

The LCSTs of PNVCL-COOH polymers were determined using a Malvern Zetasizer Nano ZS90 (Malvern Panalytical S.R.L., Malvern, UK). Samples (at a concentration of 0.5 wt.%) were incubated at different temperatures for 5 min in the temperature range between 25 and 50 °C and the cuvettes were examined to check visible turbidity. The LCST was attributed to the temperature at which a dramatic change in the shape of the autocorrelation curve was observed [39].

### 2.6. Determination of Molecular Mass

#### 2.6.1. Size Exclusion Chromatography

Samples were analyzed using a Viscotek TDA 302 (HP-SEC-TDA, Viscotek, USA), using degassed 0.3 M acetate buffer (pH = 8.0) as solvent. A volume of 100 μL of sample was injected, flow rate was maintained at 0.6 mL/min and the column and detectors temperature were kept at 25 °C. Before injection, polymer solutions were filtered through a 0.45 μm cellulose nitrate disposable membrane and the eluent was filtrated through a 16–40 μm glass filter to ensure a low light scattering noise level. A polyethyleneoxide standard (*M_W_* = 22,411, [*η*] = 0.384 dL/g, *M_w_/M_n_* = 1.03) was used to normalize the viscometer and the light scattering detectors.

#### 2.6.2. Dynamic Light Scattering

The molecular mass of the polymers was estimated using a Malvern Zetasizer Nano ZS90 (Malvern Panalytical S.R.L., Malvern, UK). All measurements were performed at 25 °C at a concentration of 0.5 wt.% in milliQ water. Each sample was analyzed three times and provided a measurement of an average hydrodynamic diameter (*D_h_*) corresponding to the position of a peak in size distribution located between 5 and 30 nm. *D_h_* standard deviation is referred to the position of the peaks and does not provide any information on peak width. For a random coil conformation, the average radius of gyration was calculated as *R_g_* = *D_h_* × 0.75. The average molecular mass was estimated from *R_g_* using the equation reported by Lau [40] (Equation (2)) and Eisele [41] (Equation (3)), respectively:

(2) $$R_{g}=2.94\times 10^{-2}M_{w}{}^{0.54}$$

(3) $$\langle s^{2}\rangle =1.77\times 10^{-18}M_{w}{}^{1.15}$$

where 〈*s*^2^〉 is the mean square radius of gyration in cm^−1^.

#### 2.6.3. Intrinsic Viscosity Measurements

The intrinsic viscosity [*η*] of PNVCL-COOH was measured at 25 °C by means of a Schott Geräte AVS/G automatic measuring apparatus and a Ubbelhode capillary viscometer, using water as a solvent. Polymer solutions and solvents were filtered prior to the analysis through 0.45 μm nitrocellulose filters (Millipore, Germany). [*η*] was calculated from the polymer concentration dependence of the reduced specific viscosity, *η_sp_*/c, according to the Huggins Equation (4):

(4) $$\eta_{red}=\frac{\eta_{sp}}{c}=\left[\eta \right]+k{\left[\eta \right]}^{2}c$$

where *k* is Huggins constant. The values of intrinsic viscosity [*η*] was calculated at infinite dilution by using two calibration lines per sample, one of which was obtained by excluding the values for the most diluted sample. [*η*] was calculated as an average of the values calculated by applying both equations, in order to provide a reasonable confidence interval for the calculated value. The corresponding average viscosimetric molecular weight (*M_η_*) of PNVCL-COOH was calculated in agreement with the Mark–Houwink–Sakurada (MHS) Equation (5).

(5) $$\left[\eta \right]=K\cdot M_{\eta }^{\alpha }$$

*K* and *a* parameters used for the calculation are reported by Kirsh [12,42] for the calculation of the molecular mass in different size range at 25 °C. The MHS equations that were used were, respectively:

(6) $$\left[\eta \right]=35\times 10^{–3}M_{\eta }^{0.57}$$

(7) $$\left[\eta \right]=38.9\times 10^{–3}M_{\eta }^{0.69}$$

## 3. Results and Discussion

PNVCL-COOH polymers were prepared via free radical polymerization as reported by Prabaharan [35]. The initial results ruled out the possibility that PNVCL-COOH was able to precipitate in diethyl ether in the conditions described by the original procedure. Accordingly, the procedure was modified in order to produce PNVCL-COOH polymers with higher molecular weight. In free radical polymerization, the degree of polymerization ${\bar{X}}_{n}$ is directly proportional to the square of the concentration of the monomer, according to the following equation:

(8) $${\bar{X}}_{n}=\alpha \frac{{\left(k_{p}\right)}^{2}}{2k_{t}v_{p}}{\left[M\right]}^{2}$$

**Figure 2:**
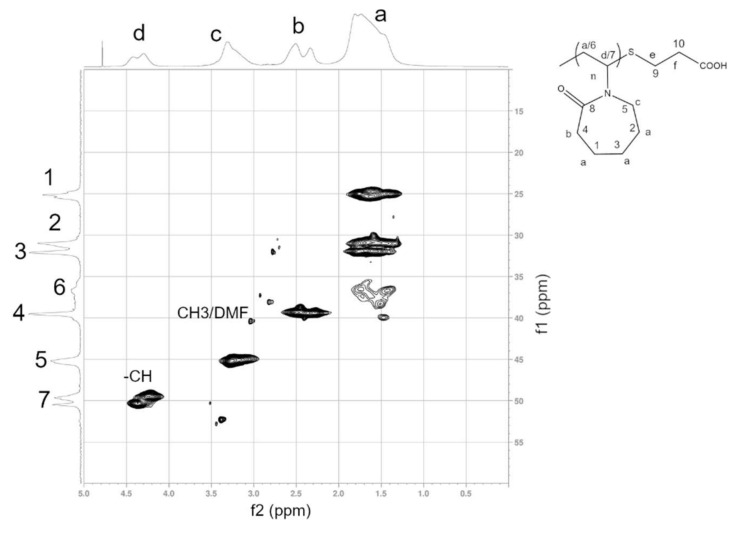
HSQC spectra of PNVCL_305.

Consequently, the preparation of PNVCL-COOH with higher molecular weight was achieved by changing the molar ratio between initiator and monomer (M/I). For this reason, PNVCL-COOH were distinguished according to the M/I molar ratio that was used for their synthesis. The M/I was, respectively, 122 (equal to that reported by Prabaharan), 244, 305, 610, 1220 and 1690. The precipitation in diethyl ether was achieved from values of M/I above 610. The elongation of the hydrophobic portion resulted in a different hydrophilic-hydrophobic balance that allowed the precipitation of PNVCL_610, PNVCL_1220 and PNVCL_1690 in diethyl ether. FRP of PNVCL is associated with a lack of control on polymer polydispersity. This could be related to the low yield of the synthesis after diethyl ether precipitation (<20%). Accordingly, the fraction of the polymer under a critical molecular mass was unable to precipitate in the solvent. The average yield of the process was raised by purifying the polymer directly with dialysis using with membrane tubings (MWCO = 1 kDa) that were compatible with DMF solutions. In this way, PNVCL-COOH polymers were obtained without diethyl ether precipitation. The main drawback of this purification method is the inability to remove eventual traces of DMF that remain in water after dialysis. In a few polymers, it was possible to identify a minor peak at 2.9 ppm (Figure 2) that demonstrates the presence of a residual DMF in freeze-dried products. The peak had been previously mistaken for the methylene group (-C-S-CH_2_) present in the terminal group [35]. This hypothesis was excluded with the utilization of HSQC-DEPT heterocorrelated spectra (see Supplementary Figure S4). All polymers exhibited different LCSTs in relation to their different molecular mass and their corresponding M/I ratio.

### 3.1. Structural Characterization of PNVCL-COOH Polymers

**Figure 3:**
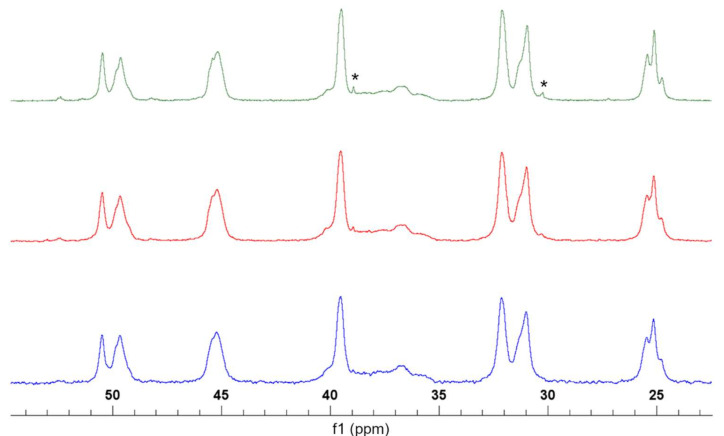
Comparison between ^13^C spectra of PNVCL_122 (green), PNVCL_305 (red) and PNVCL_1220 (blue).

NMR characterization was performed in D_2_O and d_6_-DMSO. In the ^1^H spectrum of PNVCL-COOH, four main signals were observed. The formation of the polymer was confirmed by the presence of broad signals and from the disappearance of the vinylic signals at 7.36 ppm. Similarly, amide I vibrations at 1631 and 1480 cm^−1^ (C-N stretching vibration) were observed, while the characteristic signals related to the monomer (C=C, 1658 cm^−1^, CH= and CH2=, 3000 and 3100 cm^−1^) disappeared (see Supplementary Figure S5). All ^1^H NMR PNVCL-COOH spectra exhibited peaks at 1.77 ppm (3H, -CH_2_), 2.49 ppm (2H, -COCH_2_), 3.31 (2H, -NCH_2_) and 4.36 (1H, -NCH). The HSQC correlation spectrum with ^1^H and ^13^C assignments is reported in Figure 2. ^1^H and ^13^C spectra are provided in the Supplementary Materials (Figures S1 and S3). The presence of carboxyl end groups was confirmed by analyzing PNVCL_244 and PNVCL_305 in d_6_-DMSO using a high number of acquisitions. The signals were identified in small peaks observed at about 12 ppm in both spectra (Figure S2). In FT-IR spectra, the carboxyl end groups were recognized from the presence of broad signals at 3450 cm^−1^ (Figure S5). The carboxyl group content was estimated via conductimetric titration and was found to be inversely dependent on the M/I ratio. The number of terminations for PNVCL_122, PNVCL_305, PNVCL_610, PNVCL_1220 and PNVCL 1690 were, respectively, 0.96 ± 0.25, 0.73 ± 0.17, 0.63 ± 0.14, 0.55 ± 0.12 and 0.45 ± 0.13 mmol/g. ^13^C spectra confirmed the structure of the polymers based on the previous literature [10,12] (Figure 3 and Figure S2). Heterocorrelated 2D-HSQC spectra (see Supplementary Figure S4) allowed the identification of two minor signals at 1.55/29 ppm and 2.4/38 ppm that were related to the presence of the sulphur-bonded aliphatic methylene and the methylene bonded to the carboxyl termination group. The comparison between ^13^C spectra (Figure 3) of PNVCL_122, PNVCL_244 and PNCVL_1220 in the range between 50 and 25 ppm confirmed that the signals are associated to the aliphatic portion of the termination groups. As shown, the signal intensity decreases as M/I ratio increases. This observation is in accordance with the presence of different PNVCL-COOH polymer with increasing molecular mass and increasing M/I ratio. The signals at 2.9/37 ppm were associated to CH_3_ residues of residual DMF solvent.

### 3.2. Spectroscopic Determination of LCST

**Figure 4:**
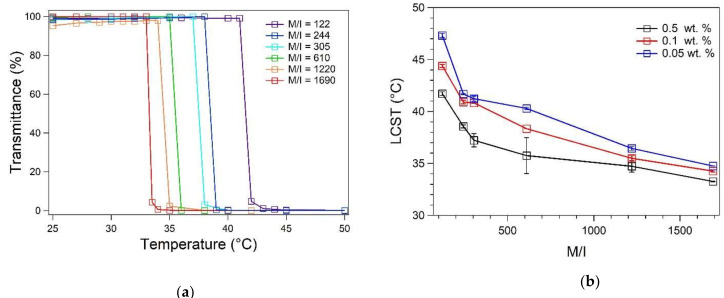

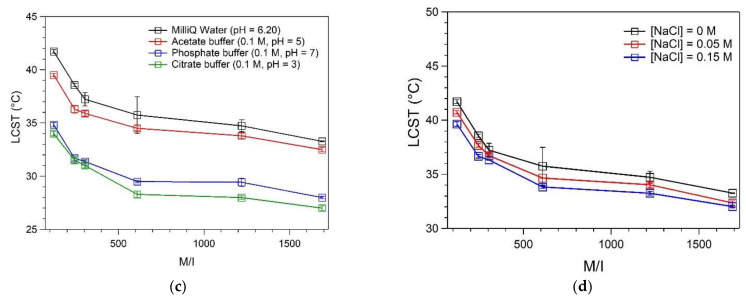
(**a**) Miscibility curves (transmittance vs. temperature) of PNVCL-COOH polymers at the concentration of 0.5 wt.% in milliQ water (pH = 6.20). (**b**) Variation of LCST reported as a function of the PNVCL-COOH concentration in milliQ water (pH = 6.20). (**c**) Variation of LCST of 0.5 wt.% PNVCL-COOH solutions associated to different type of buffers. (**d**) Variation of LCST of 0.5 wt.% PNVCL-COOH solutions (milliQ water, pH = 6.20) associated to the concentration of NaCl.

The miscibility curves were represented by plotting the transmittance of the solutions as a function of temperature (Figure 4a). LCSTs were determined from the inflection points of the sigmoidal functions that were used to fit the miscibility curves. PNVCL-COOH polymers (0.5 wt.% solutions) exhibited LCST in a range between 33 and 42 °C. The results showed that LCST diminishes in relation to the M/I ratios that were used for the synthesis of the polymers (Table 1). This molecular mass-dependent behavior is in accordance with a “classical” type I miscibility behavior. Accordingly, the increase of the hydrophobicity of the polymer results in the reduction of the LCST. The results excluded the presence of “step” transitions, that are generally observed in highly polydisperse polymers with different LCSTs. As a result, the variation of the M/I ratio provided a reliable procedure for the control of the LCST with simple FRP.

The type I behavior of PNVCL-COOH was further validated by measuring the LCSTs in the presence of salt species. The 0.5 wt.% PNVCL-COOH solutions were prepared in citrate (pH 3), acetate (pH 5) and phosphate buffers (pH 7). All buffers were prepared at the concentration of 0.1 M to compare the effect of the different ions on the LCSTs of the polymers. The results (Figure 4c) showed a strong dependence towards the types of ions dissolved in solution, as it was previously demonstrated by other studies on PNVCL polymers [4]. The results suggested that the ionic environment has a stronger effect in relation to the pH of the solution. The effect of salts was more pronounced for short chain polymers, which have a higher LCST (Figure 4c). The most evident effect was observed by dissolving PNVCL-COOH in phosphate buffer. This could be relevant from the biological point of view, since phosphate buffers solutions (e.g., PBS) are widely used in cell treatment kits [4,28,29,30,31]. During LCST transition, PNVCL-COOH undergoes a coil-to-globule transition [3,4]. Consequently, the lowering of the LCST due to the cell culture medium could result in potential cytotoxic effect due to the conformational change of the polymer. The effect of polymer concentration was assessed by measuring the LCSTs of PNVCL-COOH solutions at the concentration of 0.5, 0.1, 0.05 wt.% (Figure 4b). Normally, the LCST is associated with the point where the transmittance goes to zero [8,35,43]. However, this model was not suitable for describing the behavior of solutions at lower concentration (<0.1 wt.%). The determination of the LCST of dilute solution (<0.1 wt.%) of PNVCL has been previously reported with DLS, static light scattering and differential scanning calorimetry [4,44]. The calculation of the LCST at lower concentration was facilitated using a sigmoidal model for the interpretation of curves (Equation (1)). Dilution resulted in higher values of LCST and slower transition. Accordingly, PNVCL-COOH concentration affects the kinetics and the energy of LCST transition. Finally, the LCSTs were measured in presence of NaCl in range of concentrations between 0 and 0.15 M (Figure 4d). Results showed that LCST decreases proportionally in relation to NaCl concentration [45]. LCST decreased by about 1 °C in a physiological solution (0.9% NaCl, or 0.15 M). Consequently, pharmaceutical applications of PNVCL-COOH polymer in a physiological solution would require a polymer with a LCST of at least > 38 °C in order to avoid cytotoxic effects due to the polymer transition.

### 3.3. Scattering Determination of LCST

DLS provided lower values of LCST in relation to UV-VIS spectroscopy. The analyses of polymers with higher M/I (PNVCL_610, PNVCL_1220, PNVCL_1690) provided a direct measurement of LCST from the displacement of the autocorrelation curve due to the transition. The analyses of the other samples did not provide a direct estimation due to the small dimensions of the PNVCL-COOH macromolecules in solution. Consequently, the LCST was estimated by checking visible turbidity. The observed differences between DLS and UV-VIS measurement were related to the polydispersity of the polymers. DLS is more sensitive to the formation of aggregates in the proximity of LCST. The formation of globular aggregates at temperature inferior to LCST is related to the fraction of PNVCL-COOH polymers with higher molecular mass. Since the scattering intensity is proportional to D_h_^6^, a small fraction of polymer undergoing coil-to-globule transition is able to produce variation in the autocorrelation curve shape or position (Figure S7) [39]. Accordingly, the entity of the differences between UV-VIS and DLS measurements provided an overview on the polydispersity of PNVCL-COOH polymers. According to the results, the polymer with highest polydispersity was PNVCL_610.

### 3.4. Determination of Molecular Mass

**Figure 5:**
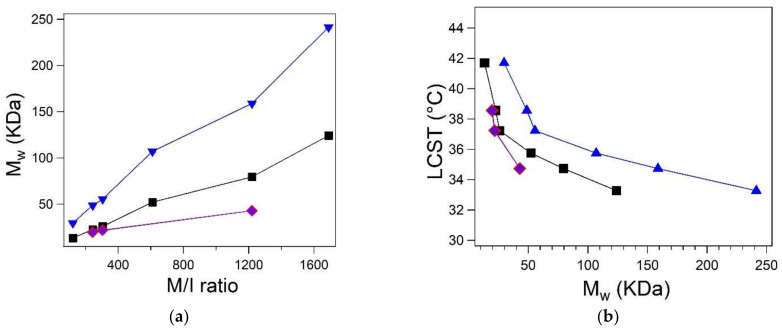
(**a**) Average molecular weight as a function of M/I ratio. (**b**) Spectroscopic LCST reported as a function of the average molecular weight. The graphics show the comparison between the values calculated using the models reported by Lau (black), Eisele (blue), and viscosimetric masses calculated using Equations (6) (red) and (7) (violet).

The molecular weight determination of polymer was carried out by SEC, DLS and viscosimetry. DLS and viscosimetry provided an estimation of the average molecular mass, while SEC did not produce any appreciable results due to the sorption of PNVCL on the column. This issue has been previously addressed for the analysis of PNVCL column in most solvents, including aqueous buffers and THF [10,46]. DLS allowed the estimation of the molecular mass according to the models described by Lau [40] and Eisele [41]. The viscosimetric molecular mass of PNVCL_244, PNVCL_305 and PNVCL_1220 mass was determined using water as a solvent at 25 °C. Samples were analyzed in dilute conditions (≤ 0.2 wt.%) in order to prevent the formation of foam. Due to the low viscosity of the sample, the differences between the run times were very small. Similarly, the values of inherent viscosity (*η_inh_*) were considered too low for the calculation of the molecular mass with the model described by Kraemer [47]. The comparison between DLS and viscosimetric measurements is reported in Table 1. The estimated molecular mass as a function of the M/I value is reported in Figure 5a and the variation of LCST as a function of the molecular mass is reported in Figure 5b. Both DLS and viscosimetric results are in line with a type I thermoresponsive polymer behavior. As the molecular mass increases, the LCST decreases. The utilization of K and a constant reported by Kirsh [12,42] demonstrated that the two models can be equally applied for PNVCL-COOH polymers in this molecular weight range (15–50 kDa). Molecular mass values obtained through viscosimetry were slightly lower than those obtained by interpreting DLS data with the model provided by Lau (Equation (2)), while the use of Eisele’s equation led to the overestimation of the molecular mass. Interestingly, it was observed that the values calculated using Lau’s equation were almost half the values obtained with Eisele’s. The bigger differences between DLS and viscosimetry data are observed with increasing molecular mass (Table 1, Figure 5a,b). Accordingly, scattering methods should be considered for the estimation of the molecular mass of PNVCL polymers with lower molecular mass, that are not suitable for viscosimetric analysis due to their low viscosity. The reported relations between LCST and *M_η_* are in accordance with the mixing behavior of aqueous PNVCL solutions reported by Meeussen [22] and Kirsh [42]. Accordingly, the small difference between the calculated LCST may be related to the different end groups of the polymers under consideration. The presence of terminations or compounds that increase the hydrophilicity of PNVCL is known to increase the LCST [27,48]. Furthermore, PNVCL-COOH polymers with a 32°C LCST have been frequently used as starting polymers for the preparation of thermoresponsive particles [7,8,9,19,35,38]. This LCST value has been previously associated to the behavior of PNVCL-COOH polymers with a molecular weight of 1 kDa by means of GPC-SEC measurements in THF [35] and never discussed in subsequent publications [7,8,9,16,17,18,19]. PNVCL_1690 shows similar properties as the LCST is close to 33°C. According to DLS measurements, PNVCL-COOH polymers require a molecular mass higher than 100 kDa in order to exhibit LCST at 32 °C. Similarly, viscosimetric analyses demonstrated that the value should be at least higher than 42 kDa, which is the value that we associated to a LCST of 34 °C. Accordingly, the importance of the molecular mass of PNVCL polymers appeared to be underestimated in studies oriented towards biological applications [7,8,9,16,17,18,19,35]. The molecular mass of the PNVCL has a central role in defining the thermoresponsive behavior of nanoparticles, microgels, gels and other biocompatible devices for drug and gene delivery. Consequently, it is essential to correctly characterize the molecular mass with precise, standardized, and reproducible methods.

### 3.5. Determination of LCST in Human Plasma 

**Figure 6:**
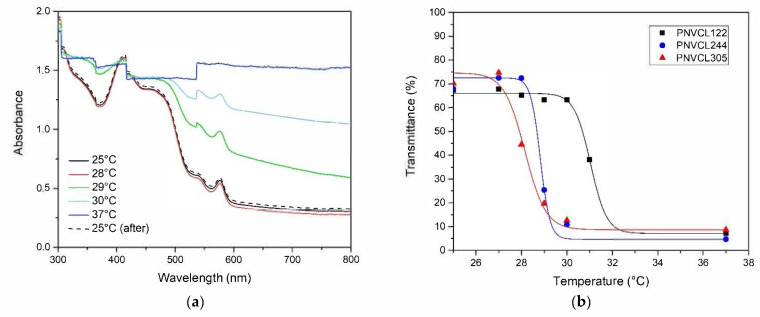
(**a**) Variation of UV-VIS spectrum of a solution of PNVCL_244 in human plasma (0.5 wt.%) with temperature. (**b**) Transmittance of PNVCL_122 (black squares), PNVCL_244 (blue circles) and PNVCL_305 (red triangles) solution in human plasma (0.5 wt.%) calculated at 800 nm reported as a function of temperature. The graph also shows the sigmoidal fitting functions that were used for the calculations of the LCST.

The critical miscibility behavior of PNVCL-COOH was observed in human plasma to evaluate possible cytotoxic effects related to the utilization of the polymer in physiological fluids. Plasma represents 55% of the blood and consists mainly of water, dissolved ions, proteins and gases. Among the main proteins, albumin, fibrinogen and globulins are present, whose functional groups give rise to the intense visible bands in the region between 200 and 600 nm [49]. In both samples analyzed, the peak at 576 nm and the shoulder at 540 nm are related to the presence of oxyhemoglobin [50] due to the hemolysis of residual erythrocytes. A first calibration of the matrix was performed on a pool of plasma realized from the union of samples taken from 12 heathy donors in a temperature range between 25 and 50 °C. The calibration excluded any spectral modification between 25 and 42 °C. Due to the complexity of the matrix, all solutions were analyzed using milliQ water as a reference. The measurements of the LCST of PNVCL_122, PNVCL_244 and PNVCL_305 were provided by the analysis of the region devoid of amino acid signals, between 600 and 800 nm (Figure 6a and Figures S8,S9). Upon heating, the absorbance signal of the spectra was shifted towards higher values due to the conformational change of the polymers. The results show that the human plasma is responsible for a significant lowering of the LCST by about 10 °C, as spectral changes were already observed at 28 °C. This is in line with the results of previous experiments, which have shown that LCST is affected by the presence of ions and proteins. By heating up the solution to 37 °C, the spectra change dramatically, and it was no longer possible to recognize any spectral information. However, the spectral properties of plasma were restored by cooling the system back to 25 °C. Accordingly, the reversibility of PNVCL-COOH transition is maintained within the plasma matrix. LCST values were calculated by fitting the transmittance vs. temperature data with a sigmoidal function (Equation (1)). Transmittance data and fitting functions are reported in Figure 6b. The comparison between the LCST of PNVCL-COOH in milliQ water and plasma are reported in Table 2. It can be concluded that the polymer is not suitable for direct utilization within the plasma at the concentration of 0.5 wt.%. However, the reversibility of the transition could be an important starting point for future developments. According to the previously reported results, the LCST can be increased by simply decreasing the chain length by modifying the M/I ratio and by diminishing polymer concentration.

## 4. Conclusions

In this work, the preparation of PNVCL-COOH linear thermoresponsive polymers with a LCST of between 33 and 42 °C was reported. The results contradict the established thesis that PNVCL has a characteristic LCST at 32 °C and exhibits a miscibility behavior similar to PNIPAM. The utilization of different ratios between monomer and initiator (M/I) allowed to lower the LCST by increasing the molecular mass of the polymer. Accordingly, the increase in molecular mass was associated with a decrease in terminal groups content that was observed in NMR spectroscopy and conductivity titration. Molecular mass characterization was approached with different method and a comparison of the results was provided. While GPC-SEC has proven to be unreliable regarding the tendency of the polymer to adsorb on the column, DLS and viscosimetry proved to be simple and effective methods for the estimation of the molecular mass in simple aqueous solution. The behavior of the polymer was described according to its concentration and in the presence of different environments, such as buffers, NaCl solutions and human plasma. The utilization of a sigmoidal model allowed to correlate the equilibrium miscibility temperature (LCST) as the inflection point of the miscibility curve. This allowed to interpret with greater precision the behavior in diluted solutions or in complex matrices. The variability associated with the LCST values in the different solutions demonstrated the importance in reporting the LCST of PNVCL polymers in relation to their concentration and molecular mass. In addition, the study demonstrates the importance of the screening of the behavior of PNVCL polymers in biologically relevant environments (plasma, PBS, physiological solution). The LCST of PNVCL polymers for biological applications should be determined within these solutions in order to prevent cytotoxic effects due to thermo-induced conformational change.

## Figures and Tables

**Table 1:** LCST, average molecular weight and LCST of the synthesized PNVCL-COOH polymers.

**M/I Ratio**	**LCST (°C)**		**Average Molecular Weight (kDa)**			
	**UV-VIS**	**DLS**	**Equation (2)**	**Equation (3)**	**Equation (6)**	**Equation (7)**
122	41.71 ± 0.17	41	13.29 ± 0.36	29.60 ± 0.99	-	-
244	38.56 ± 0.14	37	22.60 ± 0.06	48.74 ± 0.19	19.74 ± 1.12	19.56 ± 1.18
305	37.23 ± 0.63	37	25.96 ± 0.06	55.53 ± 0.17	21.87 ± 0.88	21.65 ± 0.89
610	35.75 ± 1.74	34	52.20 ± 0.36	107.0 ± 1.00	-	-
1220	34.73 ± 0.56	33.5	79.54 ± 0.25	158.9 ± 0.70	42.87 ± 1.90	42.94 ± 1.93
1690	33.27 ± 0.03	32.5	124.1 ± 0.60	241.4 ± 1.64	-	-

**Table 2:** Comparison between the LCST of PNVCL-COOH polymers (0.5 wt. %) in milliQ water and in human plasma.

**M/I Ratio**	**LCST (°C)**		
	**MilliQ Water**	**PBS (0.1 M, pH = 7)**	**Human Plasma**
122	41.71 ± 0.17	34.83 ± 0.21	31.04 ± 0.05
244	38.56 ± 0.14	31.68 ± 0.17	28.83 ± 0.11
305	37.23 ± 0.63	31.38 ± 0.14	28.15 ± 6.75

## Data Availability

The data presented in this study are available on request from the corresponding author.

## Supplementary Materials

The following are available online at https://www.mdpi.com/article/10.3390/polym13162639/s1, Figure S1: ^1^H spectrum of PNVCL_122; Figure S2: Carboxylic termination group signals in ^1^H NMR spectrum of PNVCL_305 dissolved in d_6_-DMSO; Figure S3: ^13^C spectrum of PNVCL_122; Figure S4: HSQC-DEPT spectrum of PNVCL_122; Figure S5: Comparison between NVCL and PNVCL-COOH spectrum (PNVCL_305); Figure S6 Reduced viscosity reported as a function of PNVCL_244 (black squares), PNVCL_305 (red circles) and PNVCL_1220 (blue triangles) concentration. The value of *η_red_* were calculated according to the Huggins method by using the calibration lines reported in the figure; Figure S7: Displacement of the correlation curve of PNVCL_1220 solution (0.5 wt.%) associated to LCST transition; Figure S8: Variation of UV-VIS spectrum of a solution of PNVCL_122 in human plasma (0.5 wt.%) with temperature; Figure S9: Variation of UV-VIS spectrum of a solution of PNVCL_305 in human plasma (0.5 wt.%) with temperature; Figure S10: Conductimetric titration of PNVCL_305.

## Abbreviations

[*η*]	intrinsic viscosity
AIBN	2,2′-Azobis(2-methylpropionitrile)
D_2_O	Deuterated water
*D_h_*	Hydrodynamic diameter
DLS	Dynamic light scattering
DMF	N,N′-dimethylformamide
DMSO	Dimethyl sulfoxide
FRP	Free radical polymerization
FT-IR	Fourier-transform infrared
GPC-SEC	Gel Permeation Chromatography-Size exclusion chromatography
HSQC	heteronuclear single quantum coherence
HSQC-DEPT	heteronuclear single quantum coherence-distortionless enhanced polarization transfer
LCST	lower critical solution temperature
M/I	monomer to initiator ratio
*M_n_*	Number average molecular weight
MPA	Mercaptopropionic acid
*M_w_*	weight average molecular weight
MWCO	molecular weight cut-off
*M_η_*	viscosimetric molecular weight
NHS	N-hydroxysuccinimide
NMR	nuclear magnetic resonance
NVCL	N-vinyl caprolactam
PBS	Phosphate-buffered saline
PNIPAM	poly-N-isopropylamide
PNVCL	Poly-N-Vinylcaprolactam
*R_g_*	Gyration radius
THF	Tetrahydrofuran
Tr	Transmittance
UCST	upper critical solution temperature
UV-VIS	UV-Visible
*η_inh_*	inherent viscosity
*η_red_*	reduced viscosity
*η_sp_*	specific viscosity
${\bar{\chi }}_{n}$	degree of polymerization
