# Supplementary material for: Characterization of Thermoresponsive Poly-N-Vinylcaprolactam Polymers for Biological Applications
Source: Polymers (Basel). 2021 Aug 8;13(16):2639. doi: 10.3390/polym13162639 (PMC8400179; doi:10.3390/polym13162639)
Supplement: Supplementary file 1 [file polymers-13-02639-s001.zip › polymers-1323006-supplementary.pdf]

Supporting information for

Characterization of thermoresponsive poly-N-Vinylcaprolactam for biological applications

Lorenzo Marsili[1,2,], Michele Dal Bo[1], Giorgio Eisele[3], Ivano Donati[4] Federico Berti[2] and Giuseppe Toffoli[5]*

[1] CRO National Cancer Institute; Via Franco Gallini 2, 33081 Aviano, Italy, lorenzo.marsili@phd.units.it, mdalbo@cro.it, gtoffoli@cro.it

[2] Department of Chemical and Pharmaceutical Sciences, University of Trieste, Via Licio Giorgieri 1, 34127 Trieste, Italy; fberti@units.it

[3] Ronzoni Institute; Via G. Colombo 81, 20133 Milano, Italy, eisele@cat-ronzoni.it

[4] Department of Life Sciences, University of Trieste, Via Licio Giorgieri 5, 34127 Trieste, Italy; idonati@units.it

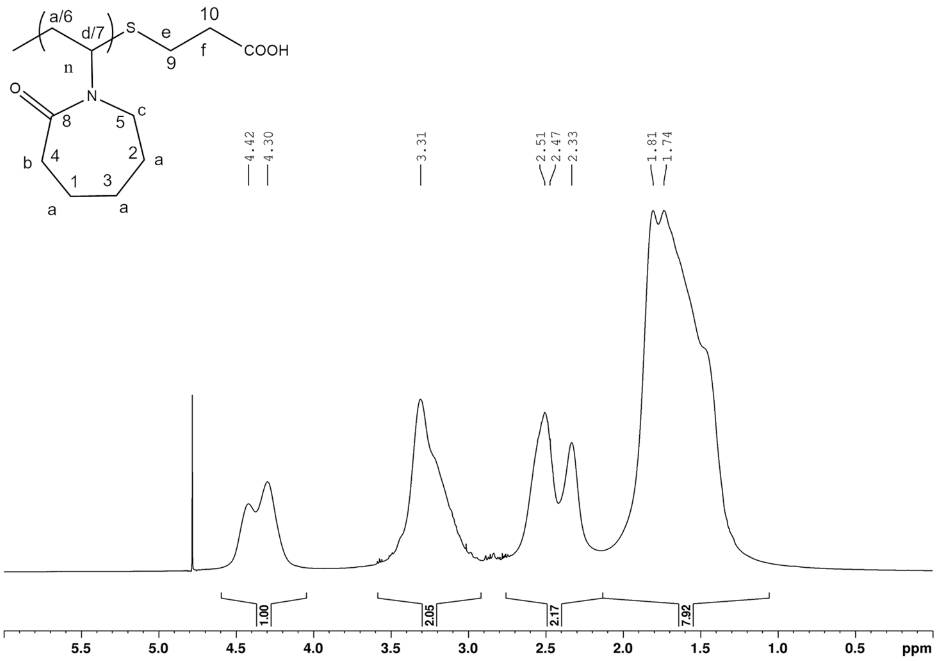

Figure S1 ^1H spectrum of PNVCL_122.

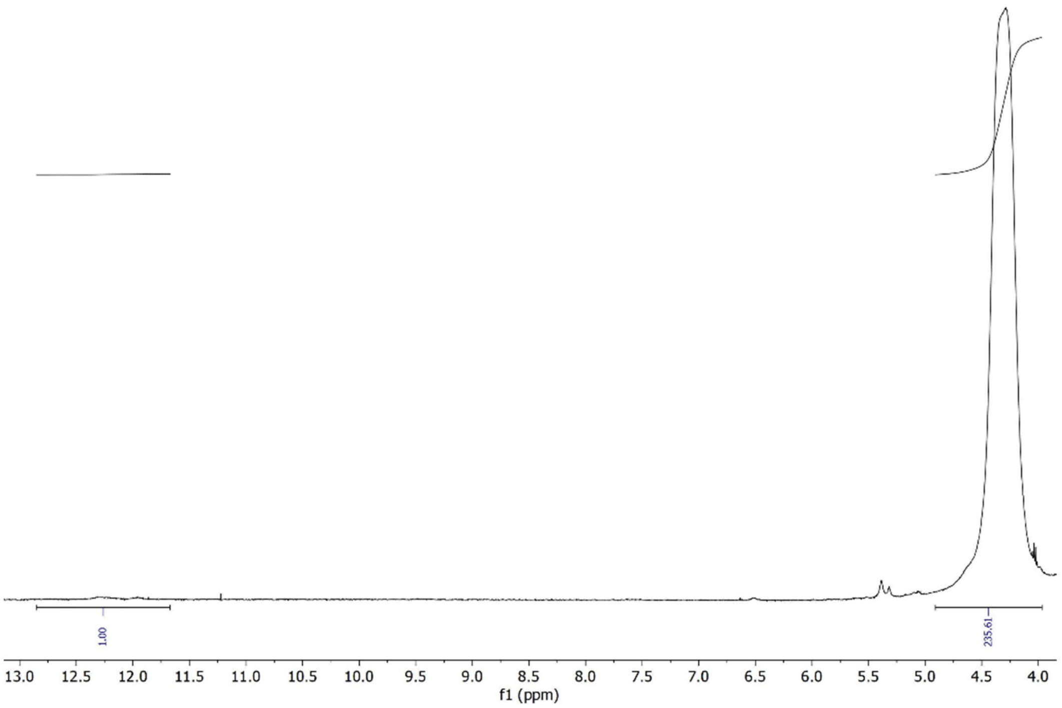

Figure S2 Carboxylic termination group signals in ^1H NMR spectrum of PNVCL_305 dissolved in d_6-DMSO.

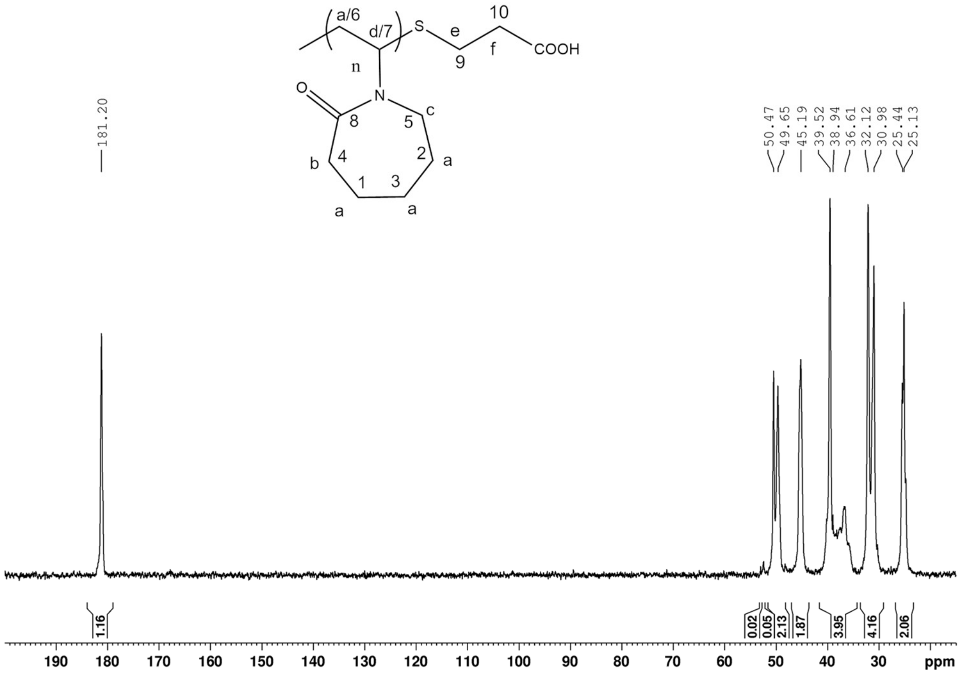

Figure S3 ^{13}C spectrum of PNVCL_122.

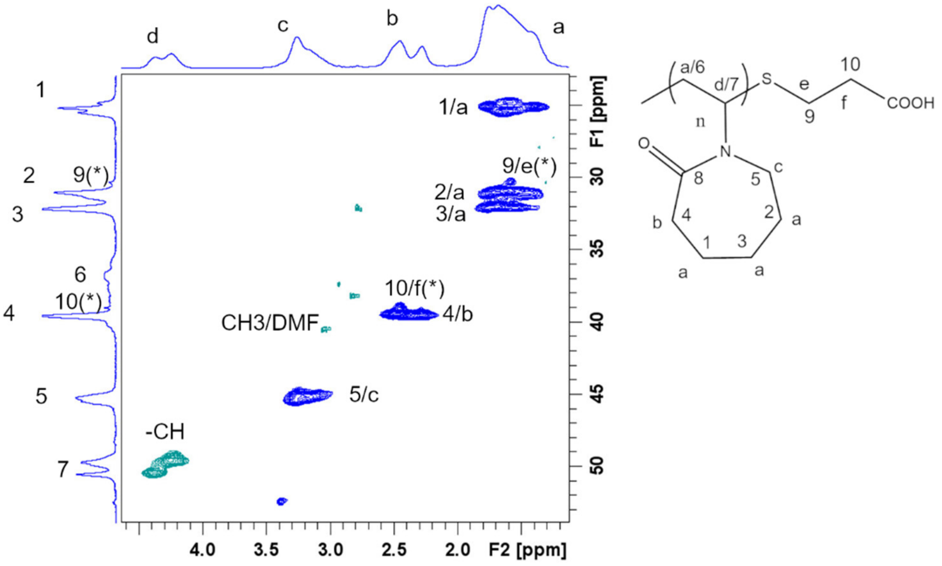

Figure S4 HSQC-DEPT spectrum of PNVCL_122.

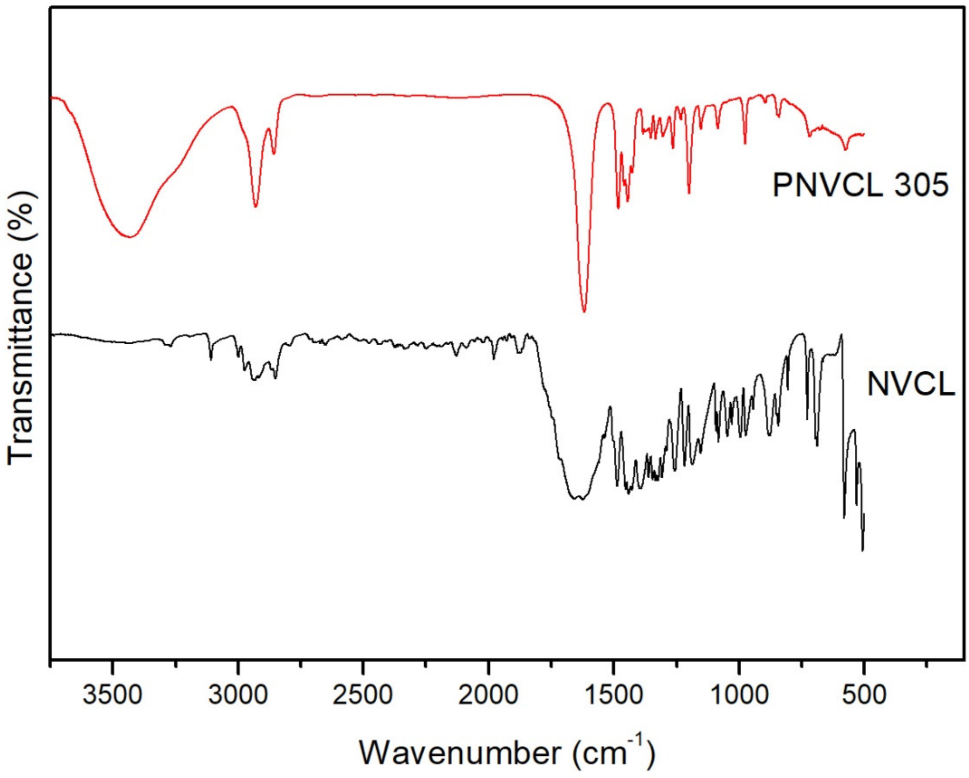

Figure S5 Comparison between NVCL and PNVCL-COOH spectrum (PNVCL_305).

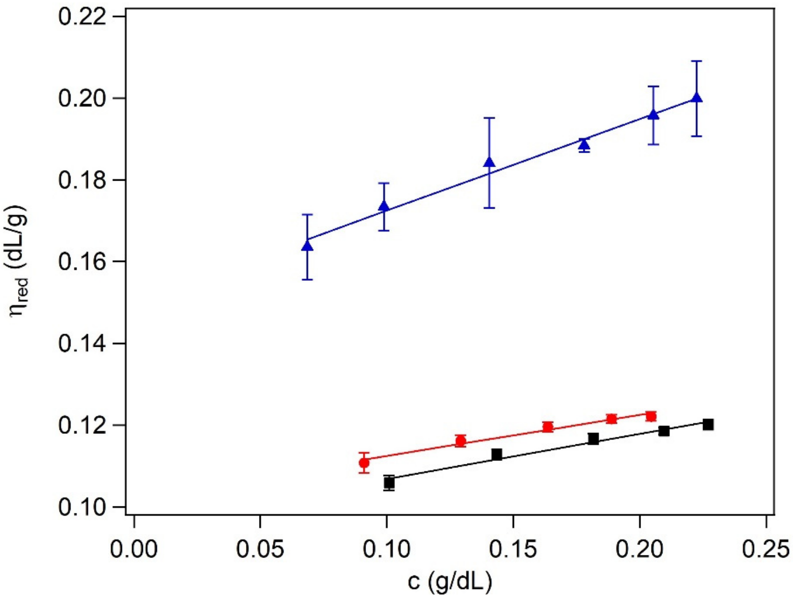

Figure S6 Reduced viscosity reported as a function of PNVCL_244 (black squares), PNVCL_305 (red circles) and PNVCL_1220 (blue triangles) concentration. The value of η_{red} were calculated according to the Huggins method by using the calibration lines reported in the figure.

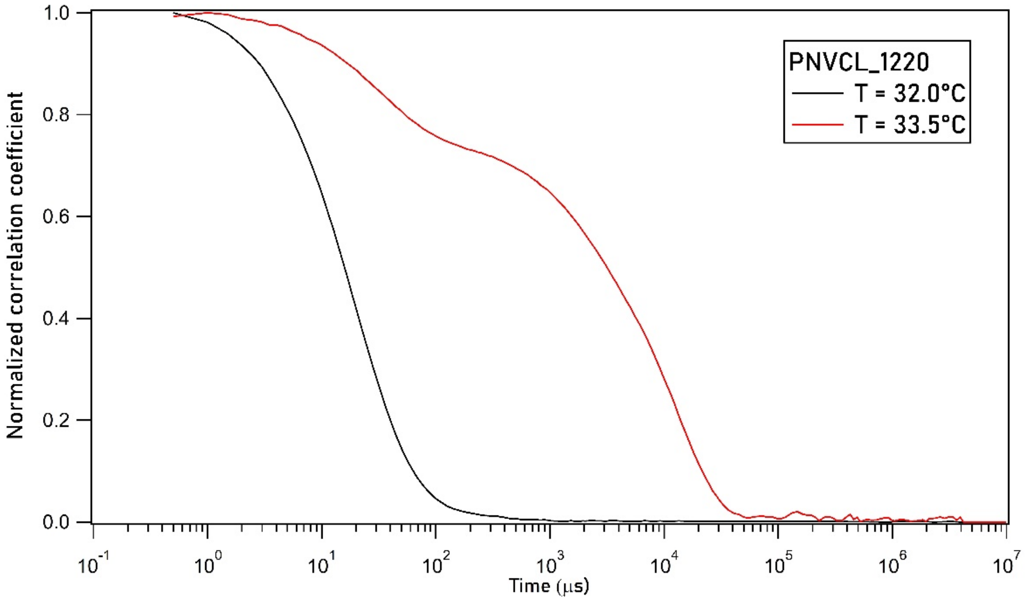

Figure S7 Displacement of the correlation curve of PNVCL_1220 solution (0.5 w%) associated to LCST transition.

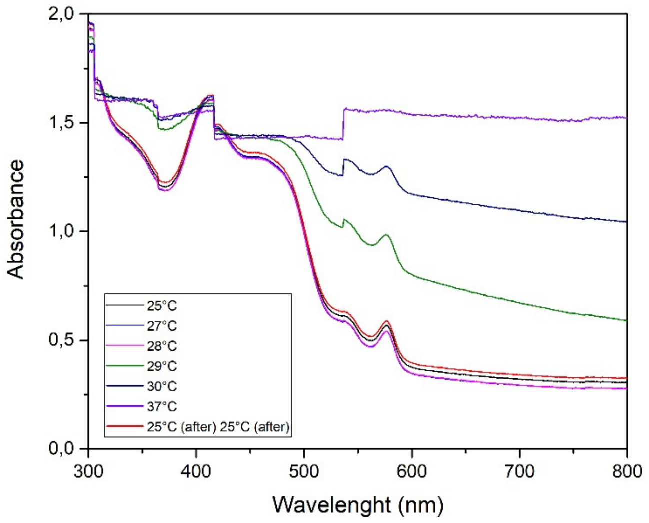

Figure S8 Variation of UV-VIS spectrum of a solution of PNVCL_244 in human plasma (5mg/ml) with temperature.

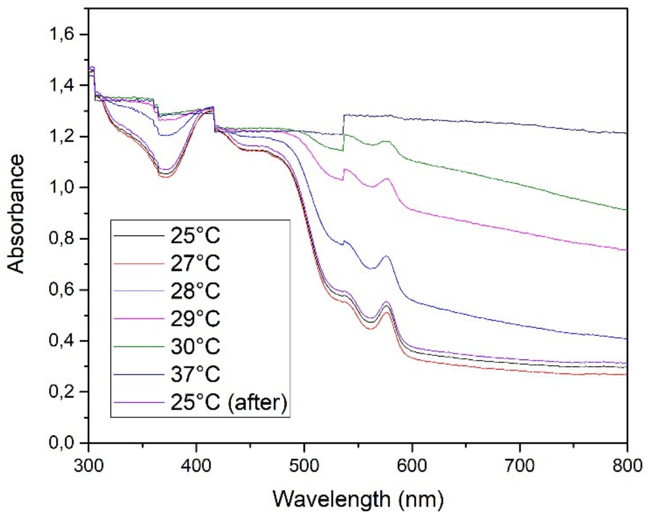

Figure S9 Variation of UV-VIS spectrum of a solution of PNVCL_244 in human plasma (5mg/ml) with temperature.

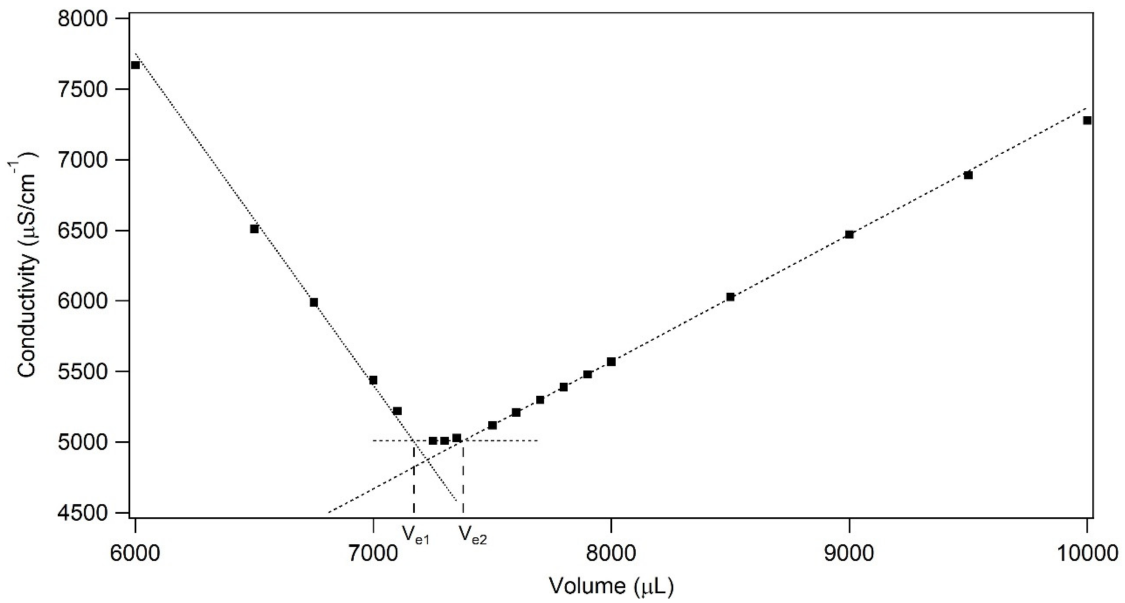

Figure S10 Conductimetric titration of PNVCL_305.
